# In the December 2012 issue

**DOI:** 10.1590/S1980-57642012DN06040001

**Published:** 2012

**Authors:** Ricardo Nitrini

**Affiliations:** Editor-in-Chief

In this issue we are publishing one review, two case reports, and eleven original papers, two of which are systematic reviews of the literature.

**Caixeta et al.** reviewed the concept that work may be a relevant etiologic factor for some mental disorders. The authors state that occupational neuropsychology is a neglected area of research and present evidence highlighting the relationship between work and cognitive dysfunction.

**Baldaçara et al.** investigated the relationship between cerebellum volume in individuals with cognitive impairment, post-traumatic stress disorders, bipolar disorders versus controls using structural MRI. The authors found that cerebellum volume was reduced in all conditions included in the study compared to controls.

**Fragoso et al.** carried out a systematic review of the literature on vitamin A and memory. Animals performed worse in the absence of dietary vitamin A but excessive amounts appeared to have a detrimental effect. The authors concluded that further studies are needed and there is currently no evidence-based data to recommend vitamin A supplementation in Alzheimer's disease (AD) and related conditions.

**Fontoura et al.** reviewed the literature on rehabilitation of language in expressive aphasias. There are a variety of techniques and theoretical approaches, which makes generalization difficult. The authors concluded that there is a need to evaluate pragmatic and social skills of communication combined with formal language tests.

**Vale et al.** proposed a new scale for evaluating memory complaints. The authors compared the performance on the scale with that on other cognitive tests and questionnaires, concluding that the Memory Complaint Scale may be a useful tool for clinicians and researchers.

**Soleman-Hernandez et al.** investigated the relationship between apathy, cognition and motor functions in patients with AD. The authors found significant association between apathy and tests of attention and walking.

**Nunes-Silva & Haase** assessed the psychometric characteristics of the Montreal Battery of Evaluation of Amusia in a sample of 150 adolescents. The authors explored the dimensional structure of the battery and concluded that almost all of the items are discriminatory and that the global score was valid and reliable. Further studies with larger Brazilian samples, including patients with amusia, are warranted.

**Vital et al.** evaluated the effects of a low intensity resistance exercise program on cognitive functions of AD patients. No significant effect of the physical exercises on cognitive performance was found. The authors concluded that more intense exercise programs should be evaluated in future studies.

**Heluani et al.** evaluated the performance of parkinsonian patients submitted to deep brain stimulation of the subthalamic nuclei on an extensive battery of cognitive tests. The authors found no difference between the pre and post-operative cognitive assessments.

**Anhoque et al.** investigated the performance of patients with clinically isolated syndrome (CIS) on a battery of neuropsychological tests. When compared with healthy controls, patients with CIS had worse performance on tests evaluating executive functions.

**Piovesana et al.** reassessed the diagnosis of AD in patients included in a high cost drug treatment program for AD. More than 50% of the patients did not fulfill diagnostic criteria for probable AD and had different demographic and cognitive performance compared with those patients fulfilling the diagnosis of probable AD. The authors considered that these data support the hypothesis of inadequate inclusion of a considerable proportion of patients in the high cost drug program for AD.

**Correia et al.** evaluated excessive diurnal sleepiness and depression in public school teachers according to number of daily shifts. Those working three shifts had more daytime sleepiness and a higher percentage of depressive symptoms.

**Almeida et al.** reported a case of subacute sclerosing encephalitis showing bilateral involvement of the basal ganglia on MRI.

**Galvão et al.** reported a case of adult-onset adrenoleukodystrophy treated for four years with the misdiagnosis of a primary psychiatric disorder. MRI findings are highlighted.

**Ricardo Nitrini** Editor-in-Chief

